# Mitochondrial Calcium Uniporter Activity Is Dispensable for MDA-MB-231 Breast Carcinoma Cell Survival

**DOI:** 10.1371/journal.pone.0096866

**Published:** 2014-05-06

**Authors:** Duane D. Hall, Yuejin Wu, Frederick E. Domann, Douglas R. Spitz, Mark E. Anderson

**Affiliations:** 1 Department of Internal Medicine, Division of Cardiovascular Medicine, Roy J. and Lucille A. Carver College of Medicine, University of Iowa, Iowa City, Iowa, United States of America; 2 Free Radical and Radiation Biology Program, Department of Radiation Oncology, Holden Comprehensive Cancer Center, Roy J. and Lucille A. Carver College of Medicine, University of Iowa, Iowa City, Iowa, United States of America; 3 Department of Molecular Physiology and Biophysics, Roy J. and Lucille A. Carver College of Medicine, University of Iowa, Iowa City, Iowa, United States of America; University of South Alabama, United States of America

## Abstract

Calcium uptake through the mitochondrial Ca^2+^ uniporter (MCU) is thought to be essential in regulating cellular signaling events, energy status, and survival. Functional dissection of the uniporter is now possible through the recent identification of the genes encoding for MCU protein complex subunits. Cancer cells exhibit many aspects of mitochondrial dysfunction associated with altered mitochondrial Ca^2+^ levels including resistance to apoptosis, increased reactive oxygen species production and decreased oxidative metabolism. We used a publically available database to determine that breast cancer patient outcomes negatively correlated with increased MCU Ca^2+^ conducting pore subunit expression and decreased MICU1 regulatory subunit expression. We hypothesized breast cancer cells may therefore be sensitive to MCU channel manipulation. We used the widely studied MDA-MB-231 breast cancer cell line to investigate whether disruption or increased activation of mitochondrial Ca^2+^ uptake with specific siRNAs and adenoviral overexpression constructs would sensitize these cells to therapy-related stress. MDA-MB-231 cells were found to contain functional MCU channels that readily respond to cellular stimulation and elicit robust AMPK phosphorylation responses to nutrient withdrawal. Surprisingly, knockdown of MCU or MICU1 did not affect reactive oxygen species production or cause significant effects on clonogenic cell survival of MDA-MB-231 cells exposed to irradiation, chemotherapeutic agents, or nutrient deprivation. Overexpression of wild type or a dominant negative mutant MCU did not affect basal cloning efficiency or ceramide-induced cell killing. In contrast, non-cancerous breast epithelial HMEC cells showed reduced survival after MCU or MICU1 knockdown. These results support the conclusion that MDA-MB-231 breast cancer cells do not rely on MCU or MICU1 activity for survival in contrast to previous findings in cells derived from cervical, colon, and prostate cancers and suggest that not all carcinomas will be sensitive to therapies targeting mitochondrial Ca^2+^ uptake mechanisms.

## Introduction

Recent genetic identification of the mitochondrial Ca^2+^ uniporter (*MCU*) [1], [2] and its associated regulatory subunit genes including *MICU1* [3]–[5] now provide molecular targets for testing the functional relevance of mitochondrial Ca^2+^ uptake in specific cell types and disease states. Mitochondrial dysfunction is common to many pathological conditions, including cancer. Cancer cells typically exhibit disease promoting characteristics linked to alterations in mitochondrial function, including increased glycolysis and reactive oxygen species (ROS) production, and resistance to apoptotic stimuli [6], [7]. Mitochondrial Ca^2+^ uptake is believed to be essential for stimulating oxidative phosphorylation [8], [9], and, if excessive, is thought to induce apoptosis through membrane permeability transition and induction of cell death pathways.

Very little is known about mitochondrial Ca^2+^ regulation or its signaling in cancer. The lack of cell membrane permeable pharmacological inhibitors of Ca^2+^ uptake has limited the ability to dissect these pathways but the recent characterization of MCU constituents has focused attention on critical components of the MCU complex. Initial evidence suggests that cancer cells reduce MCU activity for increased survival. Colon and prostate cancers overexpress a microRNA that appears to enable a pro-survival phenotype in part by targeting the MCU Ca^2+^ conducting pore subunit [10]. Furthermore, overexpression of MCU or knockdown of its auxiliary MICU1 subunit in HeLa cervical cancer cells results in constitutive mitochondrial Ca^2+^ influx and increases HeLa cell sensitivity to hydrogen peroxide and ceramide toxicity [2], [4]. However, it is not known if breast cancer cells are sensitive to mitochondrial Ca^2+^ modulation.

**Figure 1:**
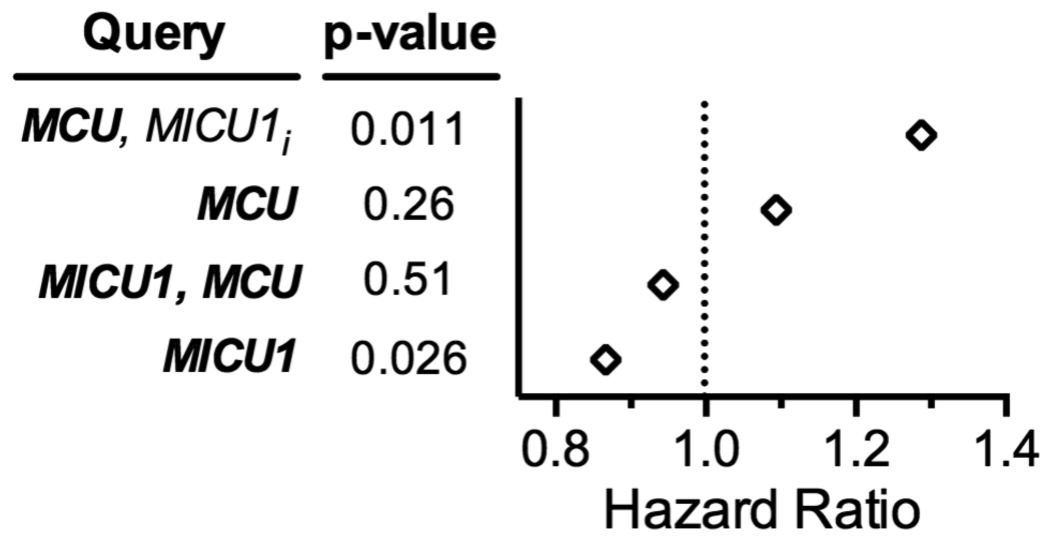
Expression of mitochondrial Ca^2+^ uniporter subunits is associated with breast cancer patient survival. Hazard ratios of queried gene expression combinations from the BreastMark mRNA survival analysis online algorithm. Patient survival was correlated to sample gene expression after genes were queried alone or in combination for overexpression (***bold***) and inverse underexpression (*not bold_i_*) and represented as hazard ratios. See Table S1 for more details including sample number, p-values, and queries for expression relationships for other uniporter subunit genes.

We queried mitochondrial Ca^2+^ uniporter subunit genes within the web based BreastMark algorithm with respect to how gene expression from breast cancer samples correlated with associated clinical outcomes [11]. A significantly poorer prognosis was associated with MCU over expression and MICU1 under expression (see Figure 1) suggesting the expression of Ca^2+^ uniporter subunits may play an important role in breast cancer biology. As triple negative breast cancers lacking estrogen, progesterone, and EGF receptors are aggressive and resistant to current available therapies, we asked if altered mitochondrial Ca^2+^ uptake could sensitize the highly aggressive triple negative MDA-MB-231 breast carcinoma cell line to therapeutically relevant stresses.

MDA-MB-231 breast cancer cells harbor mitochondrial DNA mutations [12] with decreased oxidative metabolism [13] suggesting they may utilize mitochondrial Ca^2+^ differently for energy production, and survival compared to normal cells. A recent report demonstrated that the MCU pore forming subunit of the uniporter is transcribed in MDA-MB-231 cells and may be involved in caspase-independent apoptosis [14]. We hypothesized that stimulation of MCU activity in MDA-MB-231 cells would increase mitochondrial Ca^2+^ influx leading to increased ROS levels while inhibition would reduce oxidative metabolism and limit energy availability. Both mechanisms are predicted to enhance cell killing to therapy related stress.

We modulated MCU activity in MDA-MB-231 cells through siRNA-mediated knockdown and adenoviral over expression of uniporter constituents. Mitochondrial Ca^2+^ uptake was inhibited by knockdown of *MCU* and overexpression of a dominant negative (DN) MCU mutant and enhanced through knockdown of *MICU1* and wildtype (WT) MCU overexpression. Depletion of MICU1 is known to increase basal Ca^2+^ uptake through MCU [3], [4]. Surprisingly, inhibition and activation of the MCU did not significantly alter ROS levels nor sensitize MDA-MB-231 cells to a variety of therapy relevant stresses. These results support the conclusion that MDA-MB-231 breast cancer cells function independently of MCU/MICU1 mediated mitochondrial Ca^2+^ entry, suggesting this pathway may not provide a universal therapeutic target in treating cancer.

## Materials and Methods

### BreastMark algorithm searches

The BreastMark online custom mRNA analysis algorithm tool (glados.ucd.ie/BreastMark) [11] was queried for uniporter subunit gene expression and their correlation with survival. Genes were queried separately and in combination using disease-free survival and median cutoff options. Dataset values with sample number, p-value, and hazard ratio were recorded (Table S1). A hazard ratio greater than one indicates the gene is associated with poor prognosis when overexpressed. For combined expression analysis, both parallel and inverse expression correlations were analyzed. As the order of genes within a combined analysis impacted the results of the analysis the hazard ratios are presented for the gene order as queried. Gene nomenclature within the database at the time of querying was as follows: *MCU*, *MICU1*, *CCDC109B* for *MCUB*, *CCDC90A* for *MCUR1*, *EFHA1* for *MICU2*, *EFHA2* for *MICU3*, and *C22orf32* for *EMRE*.

### Cell culture and proliferation assays

All cells were incubated in a humidified incubator with 5% CO_2_ at 37°C. The MDA-MB-231 breast cancer cell line originating from American Type Culture Collection (ATCC, HTB-26, Manassas, VA, USA) was a gift from Dr. Mary Hendrix (Northwestern University) and cultured in RPMI 1640 medium supplemented with 10% FBS and 1% penicillin/streptomycin (Gibco/Life Technologies, Grand Island, NY, U.S.A.). HeLa cells (ATCC CCL-2) were cultured in DMEM medium (Gibco) supplemented with 10% FBS and 1% penicillin/streptomycin. Primary human mammary epithelial cells (HMEC) were obtained from Lonza (East Rutherford, NJ, U.S.A.) and grown in complete MEGM medium (Lonza). For clonogenic assays, media was replenished with and without indicated drugs 24 hours prior to harvesting. For starvation experiments, culture media was replenished 24 hrs prior to confluency. Cells were washed and incubated with pre-warmed HBS buffer (140 mM NaCl, 10 mM HEPES, 2.5 mM CaCl_2_, 1 mM MgCl_2_, pH 7.4) for the indicated times. For proliferation assays, initial cell number was determined on with a Z1 Coulter Counter (Beckman Coulter, Brea, CA, USA) after >48 hours post siRNA transfection and seeded into culture dishes. Cells were grown for up to 72 hours harvested and counted. Doubling time (T_d_) of sub-confluent cultures was calculated using the equation T_d_ = 0.693t/ln(N_t_/N_0_), where N_t_ and N_0_ represent cell number at time t and time 0, respectively.

### siRNA duplexes, reverse transcription, and qPCR

Small interfering RNA (siRNA) duplexes were purchased from IDT (Integrated DNA Technologies, Iowa City, IA, U.S.A.) individually or as sets of three within TriFECTa kits and transfected into MDA-MB-231 and HeLa cells at 5 nM using 1–2 ul DharmaFECT 4 reagent (Thermo Scientific, Pittsburgh, PA, U.S.A.) per ml final volume in HMEC cultures with DharmaFECT 1. RNA was isolated using the RNAeasy Mini Plus Kit (QIAGEN) and RNA concentrations determined on a Nanodrop 2000 Spectrophotometer (Thermo Scientific, Wilmington, DE, USA). SuperScript III First Strand Synthesis SuperMix (Life Technologies) was used to make cDNA from isolated RNA as per manufacturer's directions using oligo(dT) primers. Quantitative real time PCR (qPCR) using validated PrimerPCR SYBR Green Assay human primer sets (Bio-Rad, Hercules, CA, U.S.A.) were performed using a StepOnePlus Real-Time PCR system (Applied Biosystems/Life Technologies) and quantified by the ΔΔCt method. Knockdown efficiency was consistently greater than 80% after 48 hours determined by qPCR normalized to *HPRT1* expression. More detailed information regarding siRNAs is given in Table S2.

### Adenoviral constructs

To generate adenoviral vectors for MCU overexpression and fusion with a C-terminal Myc tag, human *MCU* cDNA clone (GenBank: BC034235) was obtained from the I.M.A.G.E consortium (ID: 5296557) and subcloned into pAd5CMV-KN (University of Iowa Gene Transfer Vector Core, Iowa City, IA, U.S.A.) by PCR using the GeneArt Seamless Cloning and Assembly Kit (Life Technologies). PCR primers amplifying Myc-tagged Mcu were: forward 5′-ATA AGC TTA TGG CGG CCG CCG CAG GTA GAT CG-3′, reverse 5′-CTA CAG GTC TTC TTC GCT AAT CAG TTT CTG TTC ATC TTT TTC ACC AAT TTG TCG GAG-3′, and pAd5CMV-KN: forward 5′-GAA GAA GAC CTG TAG GAT ATC GAA TTC CTG CAG CCC-3′, reverse 5′-GCC GCC ATA AGC TTA TCG ATA CCG TCG ACC TC-3′. Dominant negative mutations in MCU that inhibit Ca^2+^ conductance (D260Q, E263Q) [1], [2] were generated with Stratagene's QuikChange site-directed mutagenesis kit. The adenoviral construct expressing mitochondrial targeted ratiometric Pericam (mt-Pericam) was generated by first subcloning ratiometric pericam-mt [15] into pAd5CMV-KN (University of Iowa Gene Transfer Vector Core) using Hind III and EcoR I. After determining some Pericam-mt remained cytosolic, we increased mitochondrial localization by fusing an additional four tandem Cox8a mitochondrial targeting repeats from pcDNA3-4mt-D3cpv [16] in frame to the N-terminus of Pericam by Hind III subcloning. Clones were confirmed by sequencing and viruses prepared by the University of Iowa Gene Transfer Vector Core.

### Mitochondrial Ca^2+^ imaging

Cells previously transfected with siRNAs for 48–72 hrs were seeded onto poly-L-lysine coated 35 mm MatTek dishes and infected with mt-Pericam adenovirus and incubated for another 48 hrs. Pericam fluorescence was measured using a Nikon Eclipse Ti microscope and NIS-Elements AR software (Nikon) in Tyrode's solution (140 mM NaCl, 10 mM Glucose, 5.4 mM KCl, 1.8 mM CaCl_2_, 1.0 mM MgCl_2_, 1.2 mM KH_2_PO_4_, 5 mM HEPES, pH 7.4 with NaOH). Cells were constantly perfused with Tyrode's solution at room temperature and Pericam was excited at 415 nm and 485 nm (ET415/30 and ET485/15, Chroma) and its emission recorded at 535 nm (T505lpxr and ET535/50, Chroma). The 485 nm/415 nm excitation ratio of the 535 nm signal was used to measure mitochondrial Ca^2+^ concentration and normalized against differences in expression level. ATP (100 µM) triggered mitochondrial Ca^2+^ concentration ([Ca^2+^]_m_) measurements were performed by rapid local perfusion (DAD Superfusion System, ALA Scientific Instruments INC, NY, USA). The mt-Pericam signals were quantified by importing data into pClamp (Molecular Devices, Sunnyvale, CA, USA) and calculating the rise in amplitude above baseline and the area under the curve for four min after ATP application.

### Clonogenic survival assays

Cells after siRNA knockdown for >48 hrs were harvested and grown in 60 mm cell culture dishes for 24 hours and, if indicated, treated for an additional 24 hours with DMSO vehicle, C2-ceramide (Enzo Life Sciences, Farmingdale, NY, USA) or paclitaxel (Sigma-Aldrich, St. Louis, MO, USA) dissolved in DMSO prior to harvesting. Cell number was determined with a Z1 Coulter Counter (Beckman Coulter, Brea, CA, USA). Survival assays were performed as previously described [17] with minor adjustments. Typically, two hundred cells were seeded in 60 mm dishes and grown at 37°C for 10–12 days (MDA-MB-231) or 12–14 days (HeLa and HMEC). Surviving colonies were fixed with 70% ethanol and stained with Coomassie blue stain, briefly washed with destain solution (40% methanol, 10% acetic acid), and rinsed with water. Dishes were scanned with an Epson Perfection 4180 Photo flatbed scanner at 800 dpi [18], [19]. Colonies were identified using ImageJ, subtracting background noise by thresholding, and measuring colony number and area by the Measure Particles option. Surviving colonies were defined as those undergoing at least 5.5 cell divisions (50 cells, average area 9.64+/−2.23×10^−5^ in^2^). To exclude colonies with less than 50 cells, a lower limit of 2.0×10^−4^ in^2^ was set. Average colony areas were consistently 10-fold larger than this value. No significant differences in colony area between groups were observed (DH, unpublished data). For the irradiation clonogenic assays, ionizing radiation was delivered at 2, 4, or 6 Gy using an X-ray source (University of Iowa, Radiation and Free Radical Research Core).

### Cell lysis, fractionation, and immunoblotting

For whole cell lysates, cells were lysed in RIPA buffer (20 mM Tris, 150 mM NaCl, 5 mM EDTA, 5 mM EGTA, 1% Triton X-100, 0.5% deoxycholate, 0.1% SDS, pH 7.4) supplemented with protease inhibitors (Mini cOmplete, Roche, Indianapolis, IN, U.S.A.) and phosphatase inhibitors (PhosSTOP, Roche). To prevent potential changes to phospho-AMPK levels during washing steps prior to lysis, plates were first cooled for 10 min by placing on pre-cooled trays on ice. Lysates were sonicated, debris pelleted by centrifugation at 16,000× *g* for 20 min at 4°C. For subcellular fractionation, cells were washed in PBS and then in HS buffer (20 mM HEPES, 250 mM sucrose, pH 7.5, protease inhibitors) prior to homogenization in cold HS buffer using 50 strokes in a Potter-Elvehjem glass Teflon homogenizer. Nuclei and cell debris were pelleted by centrifuging at 500× *g* for 5 min at 4°C. Mitochondria were separated from the cytosolic fraction by centrifuging at 6000× *g* for 10 min at 4°C. Protein concentrations were determined by BCA protein assay (Thermo Scientific) using a Tecan Infinite F200 microplate reader (San Jose, CA, USA).

For immunoblotting, proteins were run on NuPAGE 4–12% Bis-Tris gels (Life Technologies) and transferred to polyvinyl difluoride (PVDF) membranes (BioRad). Antibodies (origin, catalog number) for immunoblotting were anti-MCU (YenZym customized [20]), MICU1 (Thermo Scientific, PA5-26686), GAPDH (Cell Signaling, 2118), OxPhos complex cocktail (MitoSciences, MS604), AMPKα (Millipore, 07-350), phospho T_172_ AMPKα (Cell Signaling, 2531). Both chemiluminescence (ECL or ECL-Plus, GE Healthcare, Piscataway, NH, U.S.A.) detected by BioMax film (Kodak, Rochester, NY, U.S.A.) or infrared fluorescence (LI-COR Biosciences, Lincoln, NE, U.S.A.) visualized with the Odyssey system (LI-COR) were employed with similar results. Appropriate secondary HRP-linked antibodies for chemiluminescence were from GE Healthcare and infrared fluorescence-linked antibodies were from LI-COR.

### FACS analysis

To determine mitochondrial superoxide levels, cells were washed and resuspended in PBS with 2.5 mM CaCl_2_, 1 mM MgCl_2_, 5 mM pyruvate, and 1% BSA. Cell were labeled with 2 µM MitoSOX (Life Technologies) for 15 min at 37°C. Maximal ROS production was induced with 10 µM Antimycin A (Sigma) as a positive control. After labeling cells were filtered, placed on ice and ROS levels quantified by FACS analysis using an LSR Violet flow cytometer (University of Iowa Flow Cytometry Core) after gating and compensating for live cells with Hoechst 33258.

### Confocal microscopy

For immunofluorescence imaging, cells were grown on coverslips pre-coated with 1 µg/ml poly-L-lysine (Peptides International). Mitochondria were labeled by incubating cells with 200 nM MitoTracker (Life Technologies) at 37°C for 30 min, fixed in 4% paraformaldehyde and permeabilized with 0.1% Triton X-100 in PBS. Cells were blocked in blocking buffer (1xPBS with 2% glycerol, 50 mM ammonium chloride, 5% fetal bovine serum, and 2% donkey serum). Rabbit anti-myc and goat anti-GFP (Rockland Immunologicals) were diluted in blocking buffer at 1∶1000 and incubated overnight at 4°C. Cells were washed in PBS, reblocked and incubated with Alexa-488 conjugated donkey anti goat and Alexa-647 conjugated donkey anti rabbit secondary antibodies derived from donkey (Abcam and Jackson Immunologicals) at 1∶2000. Cells were again washed and mounted using ProLong Antifade reagent (Life Technologies). Images were acquired with a Zeiss LSM510 confocal microscope equipped with a 63× oil-immersion objective, excited with an argon laser at 488 nm filtered with NFT490 and BP505-530 Zeiss filters and HeNe laser at 543 (NFT565/BP575-615) and 633 nm (ChS1/650-730), and controlled by ZEN software (Zeiss).

### Statistical analysis

**Figure 2:**
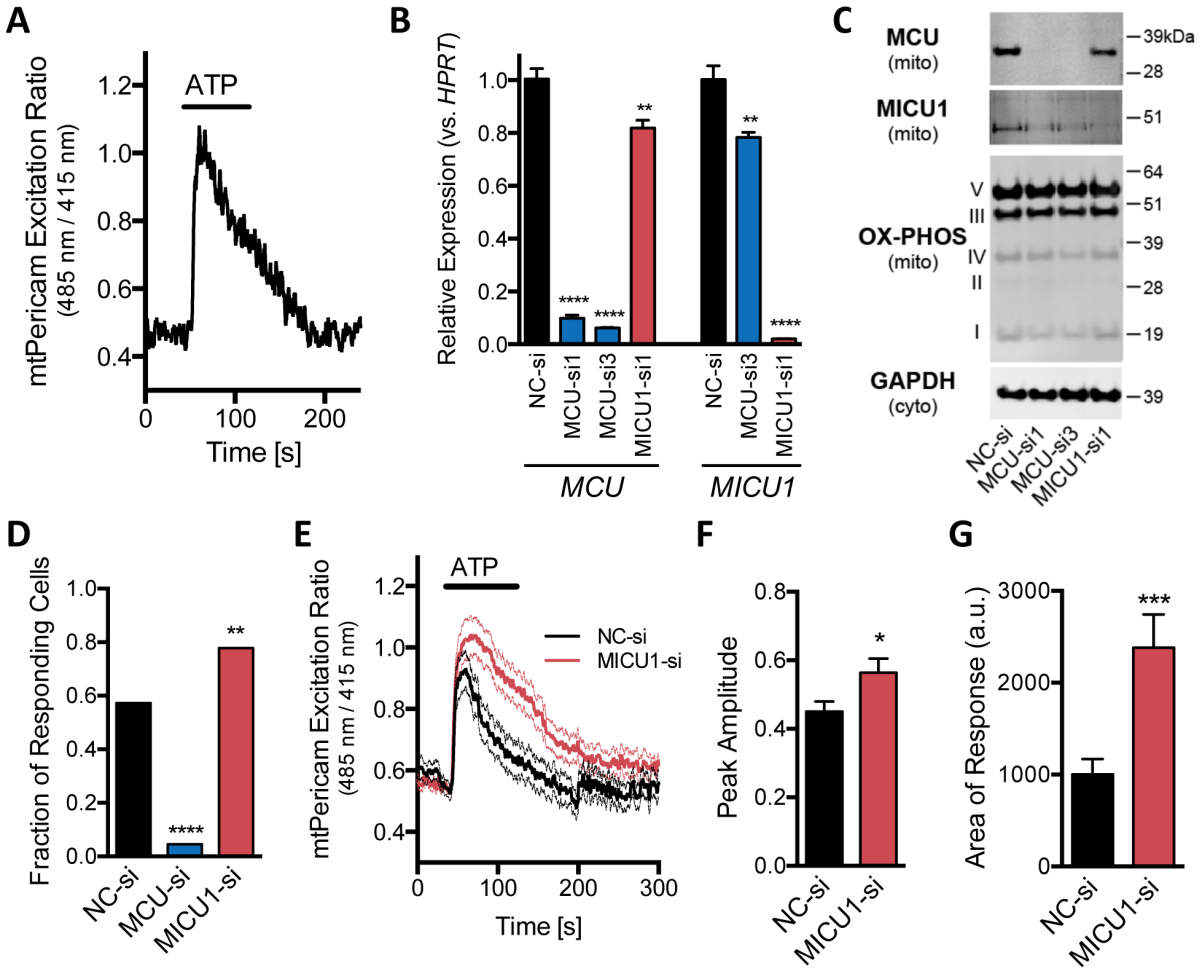
MDA-MB-231 mitochondrial Ca^2+^ influx is abrogated and enhanced by MCU and MICU1 knockdown, respectively. **A.** MDA-MB-231 mitochondria are capable of taking up Ca^2+^ in response extracellular stimulation. An example mt-Pericam recording from an adenovirally transduced cell during cellular stimulation with 100 µM ATP for the indicated duration. Mitochondrial Ca^2+^ levels were monitored from the ratio of its dual excitation absorbances (485 nm/415 nm) on emission (535 nm). **B, C.** Efficient down-regulation of MCU and MICU1 mRNA as determined by qPCR (**B**) and protein (**C**) after transient siRNA transfection. NC-si, negative control siRNA. For immunoblots (C), cells were fractionated into cytosolic and mitochondrial fractions prior to SDS-PAGE, as indicated. **D.** Fraction of mt-Pericam positive cells responding to 100 µM extracellular ATP application is significantly reduced after MCU knockdown (n = 44, p<0.0001. Fisher's exact test) and increased after MICU1 knockdown (n = 63, p<0.01) compared to NC-si (n = 129). **E.** Average mt-Pericam signals in response to 100 µM ATP as indicated in siRNA transfected cells. Averages are shown as bold lines with SEM as fine lines. **F, G.** Summary data of mt-Pericam responses calculated for peak amplitude above baseline (F) and area under of the curve (G). Data are mean +/− SEM, t-test.

Data are expressed as means +/− SEM. Experiments were performed at least three times as indicated. Significance (p<0.05) was determined with GraphPad Prism 6.0a (La Jolla, CA, USA) by students t-test when two groups were compared and ANOVA with appropriate post-hoc tests when three or more groups were compared except for Fig. 2D where Fisher's exact test was used. Significance is denoted as * for p<0.05, ** for p<0.01, *** for p<0.001, and **** for p<0.0001.

### Ethics statement

Ethical use of established commercial human cell lines, adenoviruses, and recombinant DNA was approved by University of Iowa Environmental Health Services Institutional Biosafety Committee and did not involve the use of human subjects. The authors obtained material transfer agreement rights for the use of the MDA-MB-213 cell line from American Type Culture Collection (ATCC, HTB-26, Manassas, VA, USA).

## Results

### Mitochondrial Ca^2+^ uniporter subunit gene expression negatively correlates with breast cancer survival

To assess whether breast cancers may have altered mitochondrial Ca^2+^ uptake and signaling, we interrogated the recent online BreastMark algorithm (glados.ucd.ie/BreastMark) [11] to determine whether mitochondrial Ca^2+^ uniporter subunit genes are linked to breast cancer survival. The BreastMark algorithm integrates multiple microarray experiments for which there are associated clinical data allowing correlation of gene expression with outcome. Figure 1 shows that there is a strong correlation between the hazard ratio and the expression of *MCU* and *MICU1* mRNA within breast tumors. Patients with samples overexpressing *MCU* and under expressing *MICU1* (*MCU, MICU1_i_*) have a significantly poorer prognosis (hazard ratio = 1.287, n = 1118, p = 0.011, see also Table S1) while those with *MICU1* overexpression have a better prognosis (hazard ratio = 0.8669, n = 2297, p = 0.0258). These hazard ratios are in the same range as oncogenes and tumor suppressor whose expression levels are altered in many breast tumors. For example, the tumor suppressor gene *PTEN* has a hazard ratio of 0.8931 and the oncogenes *CCND1* and *CCNE1* encoding cyclins-D1 and -E1 have hazard ratios of 1.139 and 1.374, respectively. These queries suggest that the expression ratio of the MCU Ca^2+^ channel pore subunit to the MICU1 threshold-determining subunit may be critical for breast cancer patient survival.

We would predict that increased mitochondrial Ca^2+^ uptake from MCU over expression and MICU1 under expression would increase with increased progression of breast cancer if post-translational regulatory mechanisms were otherwise unaltered (see Discussion). We set out to test this hypothesis by determining whether the commonly used MDA-MB-231 cell culture model for aggressive breast cancers exhibits dynamic mitochondrial Ca^2+^ uptake and whether its survival and response to stress is dependent on MCU activity.

### ATP-dependent increases in mitochondrial Ca^2+^ in MDA-MB-231 cells is blocked by MCU knockdown and enhanced by MICU1 depletion

MDA-MB-231 cells respond to purinergic receptor stimulation by increasing cytosolic Ca^2+^ transients [21]. We asked whether MDA-MB-231 mitochondria have functional mitochondrial Ca^2+^ uptake mechanisms and if they would buffer agonist stimulated rises in intracellular Ca^2+^. Using transduction of MDA-MB-231 cells with adenovirus expressing the mitochondrial targeted genetically encoded Ca^2+^ indicator ratiometric-pericam (mt-Pericam), we found that extracellular application of ATP translated into increased mitochondrial Ca^2+^ levels ([Ca^2+^]_m_) (Fig. 2A). Changes in [Ca^2+^]_m_ were calculated from the ratio of its Ca^2+^-insensitive 485 nm to Ca^2+^-sensitive 415 nm excitation wavelengths. The rapid response of MDA-MB-231 mitochondria to changes in cytosolic Ca^2+^ levels indicates these cells have functional MCU channels.

We confirmed that MDA-MB-231 cells express both MCU and MICU1 mRNA and protein (Fig. 2 B, C). To determine the effects of different MCU channel subunits on mitochondrial Ca^2+^ uptake, we used transient siRNA technology to effectively knockdown gene expression. Two siRNA duplexes against *MCU* and one against *MICU1* reliably reduced mRNA levels by greater than 85% by 48 hours compared to cells transfected with negative control siRNA (NC-si, Fig. 2B). Immunoblots from mitochondrial fractions show dramatically reduced levels of MCU and MICU1 protein through at least 7 days after transfection (Fig. 2 C). As both pairs of siRNAs against *MCU* effectively silenced expression, we utilized MCU-si1 in subsequent experiments, unless otherwise stated. Although a second siRNA against *MICU1* reduced mRNA levels, it did not appreciably reduce MICU1 protein levels and produced off target effects (DH and MA, unpublished data) and was therefore not used further. Interestingly, knockdown of MCU reduced MICU1 protein levels without reducing *MICU1* mRNA expression indicating potential MCU-dependent post-transcriptional feedback regulation. Reciprocal feedback did not occur, as knockdown of MICU1 did not diminish MCU protein levels.

Knockdown of MCU precluded MDA-MB-231 mitochondria from responding to extracellular ATP application. The fraction of ATP-responsive mt-Pericam positive cells decreased by more than 90% (Fig. 2D, 74/129 NC-si transfected cells responded vs. 2/44 MCU-si transfected cells). The large proportion of non-responding cells after MCU silencing indicates that MCU is essential for mitochondrial Ca^2+^ uptake. The two responding MCU-si cells displayed mt-Pericam transients that were similar to those in NC-si cells. We interpret the small fraction of responding mt-Pericam cells in the MCU-si population as those that had lost or otherwise did not contain MCU siRNA. Knockdown of MICU1 significantly increased the proportion of responding cells (Fig. 2D) and enhanced mitochondrial Ca^2+^ transients (Fig. 2 E–G) both in peak amplitude (Fig. 2F) and in the integrated area of the response (Fig. 2G). The mt-Pericam experiments show that MDA-MB-231 cells contain functional MCU channels and that MICU1 serves to temper mitochondrial Ca^2+^ uptake in response to stimuli known to increase cytosolic Ca^2+^. Our siRNA approach allows us to effectively block or enhance mitochondrial Ca^2+^ uptake and to determine the physiological role of the MCU in MDA-MB-231 cells.

### Silencing MCU and MICU1 expression has little effect on ROS production or the clonogenic survival of MDA-MB-231 cells in response to therapy-related stresses

**Figure 3:**
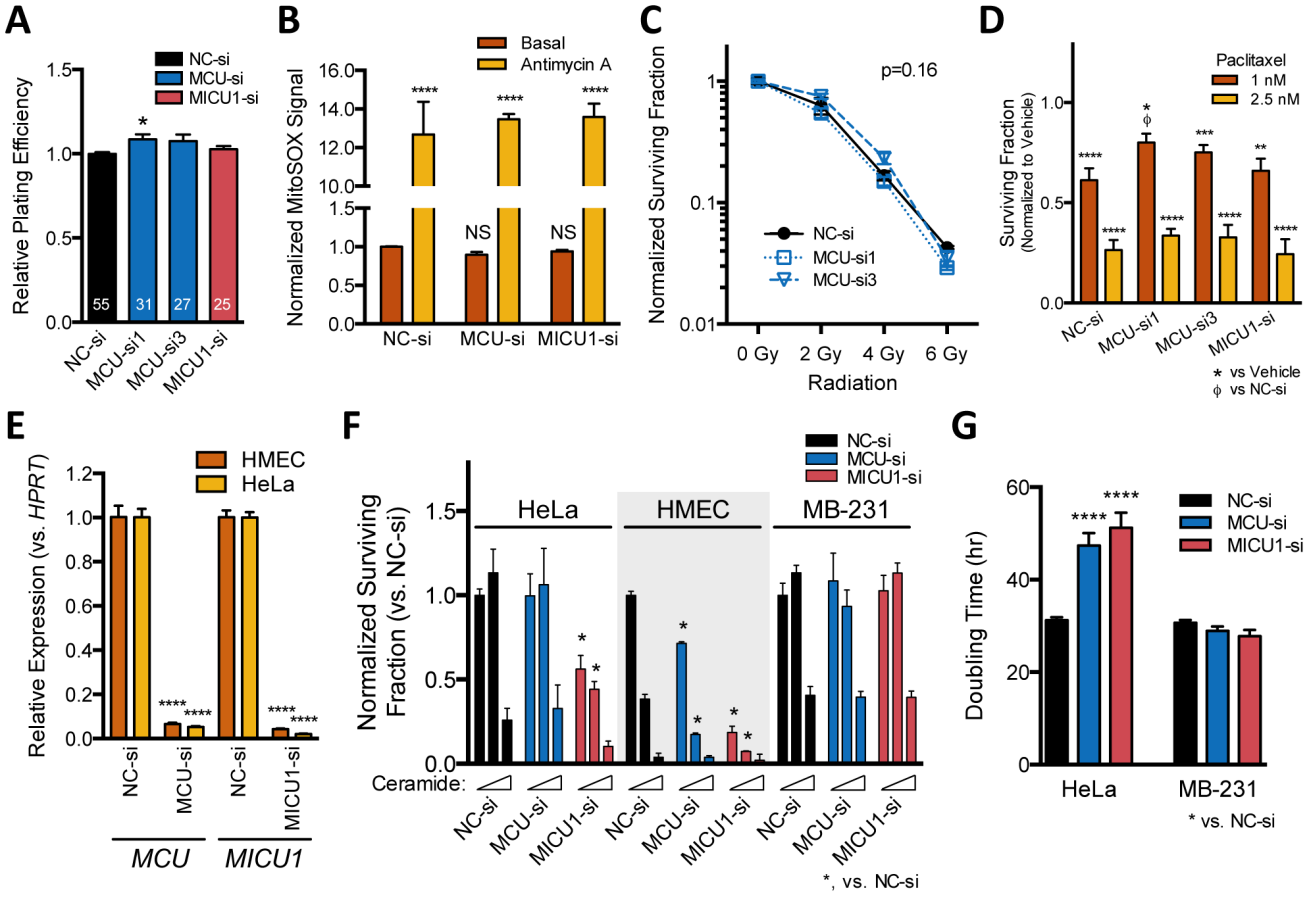
MDA-MB-231 ROS levels and sensitivity to therapy-related treatments are not mediated by MCU activity. **A.** Relative basal plating efficiency of MDA-MB-231 cells after siRNA-mediated knockdown normalized to control NC-si. (n = 25–55). **B.** Mitochondrial superoxide levels determined by FACS analysis after incubating MDA-MB-231 cells with 2 µM MitoSOX. Maximal ROS production was induced by 10 µM antimycin-A. (n = 6–7). **C–D.** Clonogenic survival assays of knockdown cells normalized to mock irradiated conditions (**C**) or DMSO vehicle (**D**). Prior to seeding, cells were irradiated with 2–6 Gy ionizing radiation (C, n = 3) or treatment for 24 hr with paclitaxel (D, n = 3–9). **E.** Knockdown efficiency of MCU and MICU1 by siRNAs in HeLa and primary HMEC cultures determined by qPCR. **F.** Clonogenic survival assays of siRNA transfected HeLa, HMEC, and MDA-MB-231 (MB-231) cells after 24 hr C2-ceramide treatment. The three bars grouped for each siRNA represent 0, 20, and 50 µM ceramide incubation, respectively (n = 3–9). The surviving fraction was determined by normalizing the number of colonies to NC-si control conditions. **G.** Proliferation rate of HeLa and MDA-MB-231 (MB-231) cells after siRNA knockdown. Data are given as means +/− SEM and significance determined by one-way (A, E, G) or two-way (B–D, F) ANOVA.

[Ca^2+^]_m_ has long been proposed to regulate mitochondrial processes [22], including flux through the electron transport chain that determines oxidative phosphorylation and contributes to ROS production. We assessed the effects of siRNAs on cellular outcomes by measuring the capacity of individual cells to survive and form colonies before and after cellular stress using clonogenic assays. The basal plating efficiency of MDA-MB-231 cells after siRNA knockdown of MCU or MICU1 was similar to that of control MDA-MB-231 cells in the absence of active treatments (Fig. 3A). Only MCU-si1, but not MCU-si3 or MICU1-si, yielded a significant (p = 0.013), but small increase of 8.6+/−2.9% in plating efficiency compared to the NC-si control. Depletion of MCU may therefore be significantly but modestly protective under basal growth conditions.

As cancer cells exhibit elevated ROS levels [6] and maintain mitochondrial oxidative capacity despite increased reliance on aerobic glycolysis [7], we examined whether manipulation of MCU activity in MDA-MB-231 cells would affect mitochondrial ROS generation and sensitize cells to treatments known to produce ROS and decrease cellular ATP levels. Knockdown of MICU1 or overexpression of MCU is reported to increase basal ROS levels and increase the AMP/ATP ratio in HeLa and endothelial cells [4]. Contrary to these findings, knockdown of MCU or MICU1 in MDA-MB-231 cells did not affect basal mitochondrial superoxide levels measured by FACS analysis with MitoSOX (Fig. 3B) suggesting MDA-MB-231 cells do not depend on MCU activity for superoxide production under basal conditions.

ROS levels can be induced by a variety of cellular stresses including ionizing radiation. Knockdown of MCU by either MCU-si1 or MCU-si3 did not alter the dose response curve of MDA-MB-231 cells to radiation (Fig. 3C. p = 0.16, ANOVA). The sensitivity of MDA-MB-231 cells to paclitaxel is increased by inhibition of glucose and hydroperoxide metabolism, suggesting that altering mitochondrial function in favor of increased steady-state levels of ROS may enhance cell killing in these cancer cells [24]. Paclitaxel is also effective at treating breast carcinomas and acts primarily by stabilizing microtubules. MCU-si1 reduced the sensitivity of MDA-MB-231 cells to 1 nM paclitaxel compared to control siRNA (p = 0.029, ANOVA), while MCU-si3 and MICU1-si were without effect (Fig. 3D).

The signaling lipid ceramide promotes Ca^2+^ leak from the endoplasmic reticulum resulting in increased mitochondrial Ca^2+^ content and apoptosis [23]. Both MCU overexpression [1] and MICU1 knockdown [4] can increase C2-ceramide toxicity in HeLa cells. To better assess the role of MCU channels in our breast cancer cell model system, we compared the basal plating efficiency and ceramide sensitivity of MDA-MB-231 cells to HeLa and primary human mammary epithelial cells (HMEC). Effective silencing of MICU1 decreased the basal cloning efficiency and surviving fraction of HeLa and HMEC cells after 20 µM ceramide treatment (Fig 3E,F). The clonogenic survival of HMECs was also reduced upon depletion of MCU. The ceramide sensitivity within MDA-MB-231 cells, however, was neither affected by MCU nor MICU1 knockdown (p = 0.13, ANOVA). Furthermore, the doubling time of HeLa cells, but not MDA-MB-231 cells, was significantly increased after acute MCU-si and MICU1-si transfection (Fig. 3G). Taken together, the capacity of MDA-MB-231 cells to grow and form colonies under either basal or oxidative stress conditions is less sensitive to MCU channel manipulation compared to primary mammary epithelial cells. HeLa cells show an intermediate phenotype, relying on MICU1 for clonogenic survival and resistance against apoptotic stimuli.

### MCU activity determines AMPK responsiveness to nutrient withdrawal without affecting starvation-induced clonogenic cell killing

**Figure 4:**
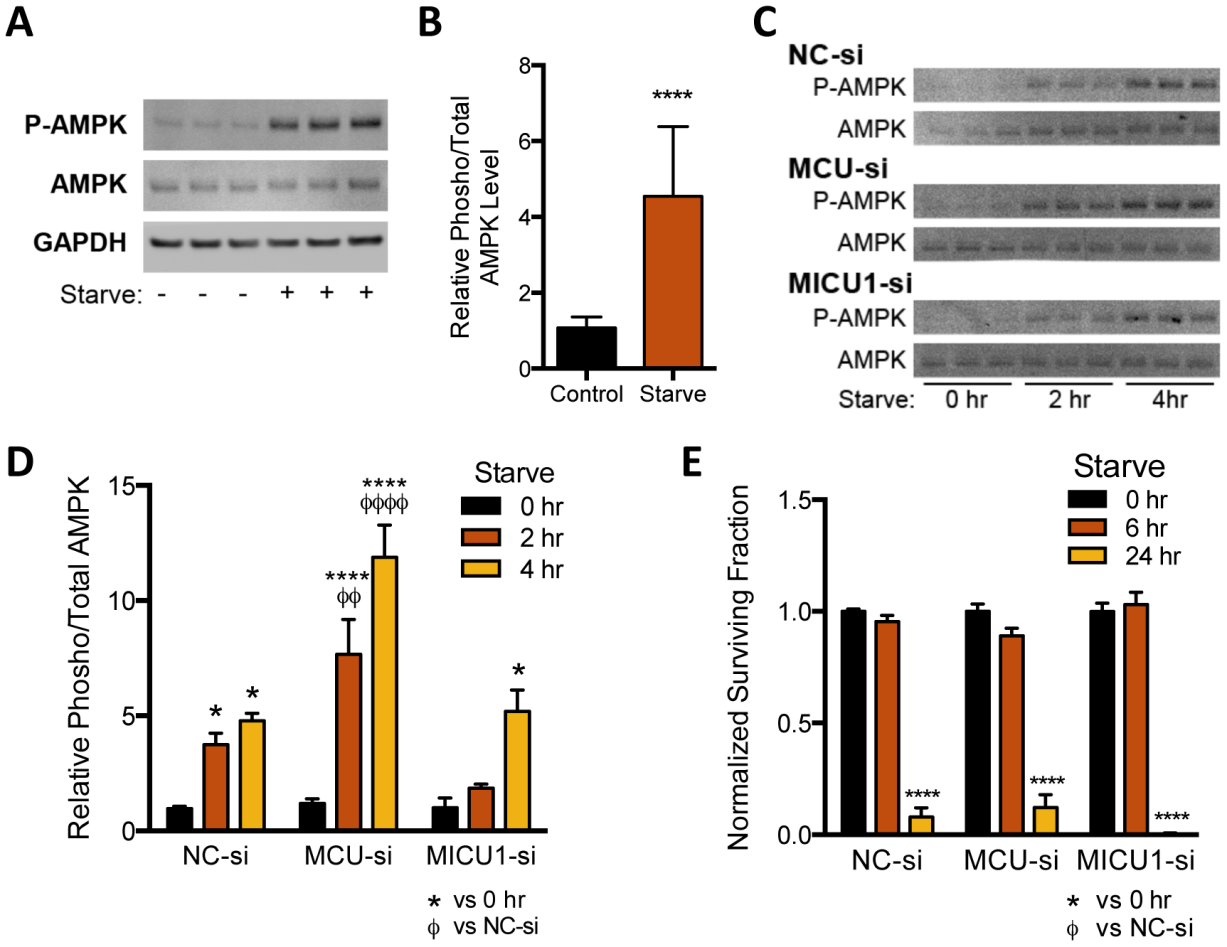
MCU activity determines AMPK activation without altering starvation-induced clonogenic cell killing. **A.** Immunoblots of lysates from cells treated for 1(HBS, Starve). **B.** Quantification of starvation response as measured by the ratio of phospho-T_172_ AMPKα (P-AMPK) to total AMPKα from immunoblots (n = 12–14, t-test). **C, D.** Similar to **A** and **B** but for siRNA transfected cells after 2–4 hrs HBS treatment. Significantly different relationships are indicated (n = 3–9, ANOVA). **E.** Clonogenic survival assay for siRNA transfected cells harvested and seeded after incubation in HBS as indicated. No significant difference between siRNA groups was found for individual starvation durations (n = 6–9, ANOVA).

As [Ca^2+^]_m_ is implicated in regulating the TCA cycle in the production of NADH to fuel oxidative phosphorylation and ATP synthesis we asked whether cellular ATP levels are dependent on MCU function. Cells respond quickly to changing energy availability through AMP-activated protein kinase (AMPK). AMPK is a critical inducer of catabolic processes by sensing even modest decreases in ATP levels with concomitant increases in AMP or ADP abundance resulting in phosphorylation of Thr172 on AMPKα. MDA-MB-231 cells display increased susceptibility to glucose deprivation conditions resulting in increased cytotoxicity and oxidative stress relative to normal cells [6]. Withdrawing nutrients from MDA-MB-231 cells by replacing growth media with HEPES buffered saline (HBS) for one hour increased phospho-AMPKα levels relative to total AMPKα, indicating that the ATP supply declined (Fig. 4 A,B). Similar to non-transfected cells, NC-si produced a ∼4–5 fold increase in phospho-AMPK levels after 2 and 4 hours HBS incubation (Fig. 4 C,D). The level of phospho-AMPK was further enhanced after MCU depletion but not significantly different in MICU1-si cells. We interpret these findings to indicate that MDA-MB-231 cells are able to sense nutrient availability and inhibition of MCU promotes activation of AMPK. Based on these results, we asked if MCU activity was critical for MDA-MB-231 cells to survive a starvation insult. Depletion of MCU or MICU1 did not alter the clonogenic survival of MDA-MB-231 cells after either 6 or 24 hrs of starvation (Fig. 4E). Accordingly, MDA-MB-231 cells utilize MCU activity to activate AMPK after nutrient withdrawal, but fail to translate that signal into a pro-survival mechanism.

### MDA-MB-231 cell survival is not altered by overexpression of WT or DN MCU

**Figure 5:**
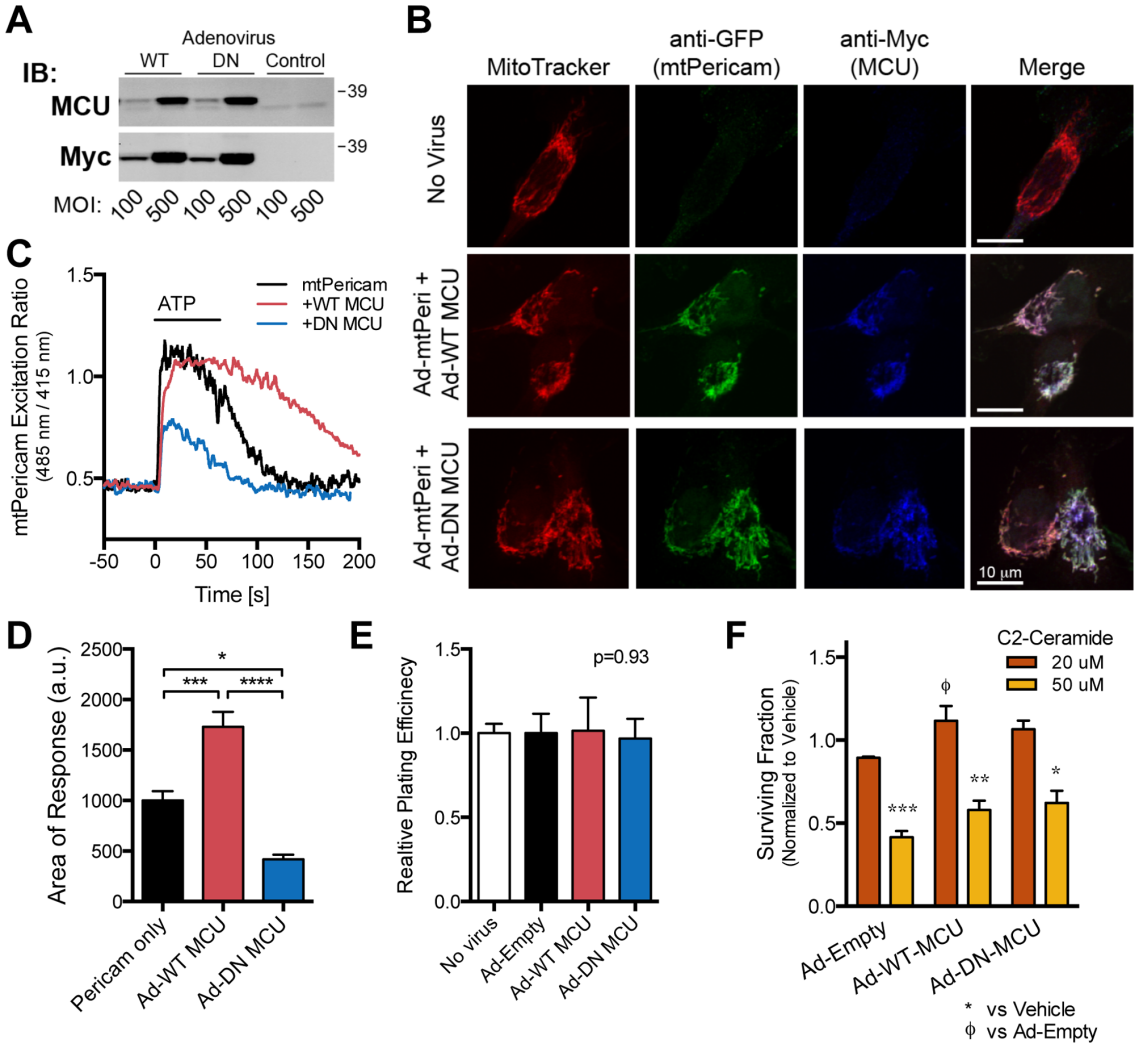
Overexpression of WT or DN MCU does not alter MDA-MB-231 cell survival. **A, B.** Adenoviruses engineered to overexpress MCU express full length, mitochondrial localized MCU. **A.** Immunoblot (IB) of MDA-MB-231 cell lysates infected with MCU-expressing adenoviruses at indicated MOI for total MCU (top) and C-terminal Myc-tag (bottom). Endogenous MCU expression can be seen as a slightly faster migrating band than tagged MCU as detected by the total anti-MCU antibody and present in control infected samples. **B.** Representative confocal immunofluorescent micorgraphs of cells with and without co-infection of MCU and mt-Pericam viruses. Mitochondrial were labeled with MitoTracker (red), fixed, and immunostained with anti-myc (blue) for overexpressed MCU-myc and anti-GFP (green) for mt-Pericam fluorescence. Colocalization of mitochondria, mt-Pericam, and overexpressed MCU is evidenced by white pixels in the merged panels. Scale bar, 10 µm. **C.** Representative mt-Pericam signal (485 nm/415 nm excitation ratio) after stimulation with 100 µM ATP as indicated with and without co-infections of MCU expressing viruses. **D.** Summary data for the integrated area of mt-Pericam responses (n = 30–86). **E.** Plating efficiency of MDA-MB-231 cells after infection with indicated adenoviruses by clonogenic assay (n = 3–9). **F.** Results of clonogenic survival assays of adenovirally transduced cells after 24 hour treatment with C2-ceramide (n = 3–9).

Although MICU1 silencing increased [Ca^2+^]_m_ in response to ATP (Fig. 2) without sensitizing MDA-MB-231 cells to therapy-related and starvation treatments (Figs. 3, 4), we wanted to be confident that MCU activity in MDA-MB-231 cells does not regulate survival at baseline or during stress. Overexpression of MCU increases [Ca^2+^]_m_ in response to histamine stimulation and increases ceramide-induced toxicity in HeLa cells [1]. We therefore generated adenoviruses to overexpress WT MCU or a DN form of MCU by mutating D_260_ and E_263_ residues to glutamine causing loss of Ca^2+^ conductance [1]. Viruses expressed full length C-terminal myc-tagged MCU that migrated just above endogenous MCU and localized to mitochondria (Fig. 5 A, B). Co-transduction of MCU expressing viruses with mt-Pericam virus shows that WT-MCU increases ATP-induced increases in [Ca^2+^]_m_ while DN-MCU decreases [Ca^2+^]_m_ (Fig. 5 C, D). The integrated area of the mtPericam signal is significantly larger after WT MCU expression and lower after DN-MCU expression compared to mt-Pericam alone. Similar to the siRNA results, overexpression of WT or DN MCU did not alter the basal plating efficiency (Fig. 5E) or clonogenic survival after ceramide treatment (Fig. 5F). Taken together, our results demonstrate that inhibiting MCU activity through MCU siRNAs or DN-MCU adenoviruses or enhancing mitochondrial Ca^2+^ influx through MICU1 siRNA or WT-MCU overexpression does not affect survival mechanisms in MDA-MB-231 cells.

## Discussion

Despite the strong indication that the expression of the *MCU* and *MICU1* mitochondrial Ca^2+^ uniporter subunit genes within breast tumors would be valuable prognostic indicators for patient survival (Fig. 1), such a relationship did not manifest itself in our cell culture model using the aggressive and extensively studied MDA-MB-231 breast cancer cell line. We were able to effectively inhibit mitochondrial Ca^2+^ uptake by silencing endogenous *MCU* or by overexpression of a Ca^2+^ impermeant mutant of MCU and enhance influx by knocking down *MICU1* or overexpressing WT MCU. However, in contrast to our findings in normal breast epithelial and HeLa cells, siRNA and adenoviral manipulations of uniporter activity did not readily translate into physiological phenotypes in MDA-MB-231 cells. We found that modulation of mitochondrial Ca^2+^ uniporter activity only subtly impinges on overall MDA-MB-231 cell survival and sensitivity to therapy-related stresses that rely on mitochondrial function. The lack of striking changes in the sensitivity of MDA-MB-231 cells to different cellular stresses including cytotoxicity, ionizing radiation, chemotherapy, and starvation suggest that these cells primarily utilize MCU-independent mechanisms for survival.

Our current results expand upon those from an initial report by Curry et al. who found breast tumors of basal origin have higher relative *MCU* mRNA expression compared to other tumor subtypes [14]. Basal-like MDA-MB-231 cells with MCU knockdown exhibited increased apoptosis in response to ionomycin treatment that was found to act through a Ca^2+^-dependent, but caspase-independent cell death pathway. Our attempts to challenge MDA-MB-231 cells with a variety of cellular stress protocols suggest that survival and colony formation is not exquisitely sensitive to MCU modulation. We observed a modest increase in basal plating efficiency and protection from paclitaxel treatment with one, but not another, specific MCU siRNA and not after MICU1 knockdown (Fig. 3). We interpret our findings to indicate MDA-MB-231 cell survival mechanisms are not dependent or only minimally dependent on MCU-mediated mitochondrial Ca^2+^ uptake.

We were surprised that MICU1 knockdown did not show a clear phenotype in MDA-MB-231 cells as it did in normal HMEC and HeLa cells similar to previous findings [3], [4]. In agreement with our observations that the clonogenic survival in response to ceramide depends on MICU1 in HMEC and HeLa cells, knockdown of MICU1 in HeLa and endothelial cells increases basal [Ca^2+^]_m_, prolongs [Ca^2+^]_m_ transients, increases ROS production, increases the sensitivity to apoptotic stimuli, and decreases migration [3], [4]. The increase in ceramide toxicity could be rescued by adenoviral expression of ROS scavenging enzymes showing an interdependence between elevated [Ca^2+^]_m_, ROS, and apoptotic agents. Although we clearly reduced MICU1 expression in MDA-MB-231 cells with our siRNA approach and facilitated mitochondrial Ca^2+^ uptake in response to cellular stimulation, we did not observe changes to basal mitochondrial Ca^2+^ concentration as measured by mt-Pericam, ROS levels, sensitivity to an array of stressors including ceramide treatment (Figs. 2–4) or changes in migration rate (DH, unpublished data). The lack of phenotypic effects from MICU1 knockdown in our clonogenic assays may therefore be due to a deficient functional platform (i.e. unaltered basal [Ca^2+^]_m_ and/or ROS levels) for these agents to elicit their effects. MDA-MB-231 cells appear to uncouple mitochondrial Ca^2+^ transport from other mitochondrial-responsive mechanisms as has been determined for other cell types.

MCU and MICU1 were the first of several uniporter channel subunits identified including MCUB [27], MCUR1 [28], EMRE [29], MICU2 [30], [31] and possibly MICU3 [30]. How the macromolecular uniporter channel complex is expressed, assembled, and post-translationally regulated in different cells types and disease conditions remains to be discerned. There is evidence that the assembly of the MCU complex proteins is highly interdependent. We see that MCU knockdown decreases MICU1 protein abundance without affecting MICU1 mRNA levels but MICU1 silencing had no effect on MCU levels (Fig. 2B, C). MCU knockout in HEK cells has been reported to reduce MCUB and EMRE protein levels, but not MICU1 abundance [29]. MICU1 knockdown causes the concomitant decrease in MICU2 protein levels [29], [30], but not vice versa, uncovering a likely indirect role of MICU1 in eliminating the lower threshold of Ca^2+^ conductance through MCU [31].

Although MDA-MB-231 cell survival phenotypes fail to respond to MCU modulation, it does not exclude the possibility that other breast tumor cells may be sensitive to MCU inhibition. Searching the BreastMark algorithm for other uniporter subunit genes as performed in Figure 1 further suggests that if protein abundance and activities of these subunits correlates with mRNA levels within tumor samples, the resulting effect on MCU activity would inversely correlate with patient outcome (Table S1). Both MCUB and EMRE have favorable hazard ratios similar to MICU1, while MCUR1 has a hazard ratio of 1.287. These values inversely correlate with the proposed functions of MCUB as an endogenous inhibitory subunit in MCU/MCUB heteromeric channels, EMRE as an essential factor for MCU to assemble with MICU1, and MCUR1 as a necessary member for MCU-dependent Ca^2+^ influx. Interestingly, combinatorial searches for MCU, MICU1, and MCUB co-expression patterns yields a broader hazard ratio range to more beneficial or poorer values. We have begun to investigate the effects of MCUB silencing in MDA-MB-231 cells and, similar to MCU and MICU1, MCUB siRNAs have little effect on the clonogenic survival at baseline or after therapy-related cellular stress. In the future, it will be interesting to determine whether the predicted increase in MCU activity that we see in gene expression datasets is indeed causative for poor patient prognosis in breast cancer patients.

The hypothesized importance of MCU activity in mitochondrial metabolism, ROS production, and adaptation to stress has been supported by our findings in HMEC and HeLa cells and in experiments using cells derived from cervical [1]–[4], prostate, and colon cancers [10] and involved in pathophysiological responses such those occurring during cardiac ischemia reperfusion injury [20] and neuronal excito-toxicity [25]. MDA-MB-231 cells appear to have lost these MCU-mediated mechanisms and indicate that the requirement for mitochondrial Ca^2+^ uptake through MCU is not universal. In support of this notion, Pan, et al. recently reported that MCU knockout mice lack a robust phenotype [26]. Similar to our observations, MEFs isolated from these knockout animals failed to show differences in apoptotic responses to a variety of agents, including ceramide, compared to WT mice. How MDA-MB-231 cells and MCU knockout mice have acquired the ability to adapt and survive independent of the mitochondrial Ca^2+^ uniporter remains to be understood. Compensatory mechanisms may exist in these systems for maintaining basal mitochondrial Ca^2+^ levels or within the downstream mitochondrial Ca^2+^ dependent signaling processes.

## Conclusions

In summary, we find that the viability and proliferative capacity of MDA-MB-231 cells does not depend on mitochondrial Ca^2+^ influx through the uniporter complex. Inhibition and activation of MCU activity did not affect basal plating efficiency nor clonogenic survival in response to irradiation, the chemotherapy agent paclitaxel, the ER stress and apoptosis-inducing lipid ceramide, or nutrient withdrawal. Our data show targeting MCU channels may not be a reliable therapeutic option in treating all cancers.

## Supporting Information

Table S1 **BreastMark algorithm queries for mitochondrial Ca^2+^ uniporter subunit gene expression correlated to patient outcome.**
(DOC)

Table S2 **Details of siRNA duplexes used in this study.**
(DOC)
