# Supplementary material for: Mitochondrial Calcium Uniporter Activity Is Dispensable for MDA-MB-231 Breast Carcinoma Cell Survival
Source: PLoS One. 2014 May 6;9(5):e96866. doi: 10.1371/journal.pone.0096866 (PMC4011874; doi:10.1371/journal.pone.0096866)
Supplement: Table S1 — BreastMark algorithm queries for mitochondrial Ca2+ uniporter subunit gene expression correlated to patient outcome. (DOC) [file pone.0096866.s001.doc]

| **Table S1. BreastMark^a^ algorithm queries for mitochondrial Ca^2+^ uniporter subunit gene expression correlated to patient outcome.** | | | | | | |
| --- | --- | --- | --- | --- | --- | --- |
| **Gene query^b^** | | |  | | | |
| **First** | **Second** | **Third** | **Hazard Ratio** | **N=** | **#events** | **p-value^c^** |
| ***MICU1*** | ***-*** | ***-*** | **0.8669** | **2297** | **979** | **0.0258** |
| *MCU* | *-* | *-* | 1.094 | 1178 | 635 | 0.26 |
| ***MCUB (CCDC109B)*** | ***-*** | ***-*** | **0.8629** | **2467** | **1008** | **0.0193** |
| *EMRE (C22orf32)* | *-* | *-* | 0.8881 | 1178 | 625 | 0.139 |
| *MICU2 (EFHA1)* | ***-*** | ***-*** | 0.9443 | 2297 | 979 | 0.37 |
| *MICU3 (EFHA2)* | *-* | *-* | 0.9548 | 924 | 543 | 0.59 |
| ***MCUR1 (CCDC90A)*** | ***-*** | ***-*** | **1.287** | **1771** | **705** | **0.00808** |
| *MCU* | *MICU1* | ***-*** | 0.9431 | 1118 | 607 | 0.51 |
| ***MCU*** | ***MICU1(i)*** | *-* | **1.287** | **1118** | **607** | **0.0113** |
| *MICU1* | *MCU* | ***-*** | 0.9431 | 1118 | 607 | 0.51 |
| *MICU1* | *MCU(i)* | *-* | 0.9738 | 1118 | 607 | 0.8 |
| ***MICU1*** | ***MCUB*** | ***-*** | **0.7671** | **2112** | **866** | **0.00109** |
| *MCU* | *MCUB* | *-* | 0.8477 | 1069 | 537 | 0.1016 |
| ***MCU*** | ***MCUB(i)*** | ***-*** | **1.294** | **1069** | **537** | **0.0086** |
| ***MICU1*** | ***MCU(i)*** | ***MCUB*** | **0.653** | **2297** | **979** | **6.22E-09** |
| ***MCU*** | ***MICU1*** | ***MCUB*** | **0.791** | **2112** | **866** | **0.00868** |
| ***MCU*** | ***MICU1(i)*** | ***MCUB(i)*** | **1.738** | **1178** | **635** | **1.56E-08** |
| *PTEN* | ***-*** | ***-*** | 0.8931 | 2652 | 1121 | 0.05876 |
| *CCND1* | *-* | *-* | 1.105 | 2357 | 1007 | 0.1149 |
| ***CCNE1*** | ***-*** | ***-*** | **1.374** | **2357** | **1007** | **4.45E-07** |
| ^a^ glados.ucd.ie/BreastMark. | |  |  |  |  |  |
| ^b^ Order in which gene expression was searched. | | | | | | |
| ^c^ Queries with significance of p<0.05 are bolded. | | | | | | |
| (i), Inversely correlated underexpressed genes within same sample. | | | | | | |
