# Supplementary material for: Mitochondrial Calcium Uniporter Activity Is Dispensable for MDA-MB-231 Breast Carcinoma Cell Survival
Source: PLoS One. 2014 May 6;9(5):e96866. doi: 10.1371/journal.pone.0096866 (PMC4011874; doi:10.1371/journal.pone.0096866)
Supplement: Table S2 — Details of siRNA duplexes used in this study. (DOC) [file pone.0096866.s002.doc]

| **Table S2. Details of siRNA duplexes used in this study.** | | | |
| --- | --- | --- | --- |
| **siRNA** | **Oligo** | **Sequence** | **IDT Catalog Number** |
| NC-si | Sense | CGUUAAUCGCGUAUAAUACGCGUdAdT | Included in TriFECTa Kit |
|  | Antisense | AUACGCGUAUUAUAVHVHAUUAACGAC |  |
| MCU-si1 | Sense | CCUAGAGAAAUACAAUCAACUCAdAdG | HSC.RNAI.N138357.12.1 |
|  | Antisense | CUUGAGUUGAUUGUAUUUCUCUAGGUC |  |
| MCU-si2 | Sense | AGACUAGAGGAUCUCAAAGAGCAdGdC | HSC.RNAI.N138357.12.2 |
|  | Antisense | GCUGCUCUUUGAGAUCCUCUAGUCUUU |  |
| MCU-si3 | Sense | GCAAUGUACCUUCUGGAACAAUAdAdA | HSC.RNAI.N138357.12.3 |
|  | Antisense | UUUAUUGUUCCAGAAGGUACAUUGCUU |  |
| MICU1-si1 | Sense | CGAACUGAGCAAUAAGGAAUUUGdTdT | HSC.RNAI.N006077.12.1 |
|  | Antisense | AACAAAUUCCUUAUUGCUCAGUUCGCC |  |
| MICU1-si2 | Sense | GCCACAAGUAGGGAAGAUAUCUGdGdC |  |
|  | Antisense | GCCAGAUAUCUUCCCUACUUGUGGCUU | HSC.RNAI.N006077.12.2 |
